# PILOTING COMMUNICATION TOOLS TO ENHANCE RESIDENT/FAMILY ENGAGEMENT IN NURSING HOMES CARE CONFERENCES

**DOI:** 10.1093/geroni/igad104.2887

**Published:** 2023-12-21

**Authors:** Jingxi Li, Alexa Roscizewski, Terra Warthen, Elizabeth Cox, Tonya Roberts

**Affiliations:** University of Wisconsin-Madison, Madison, Wisconsin, United States; University of Wisconsin-Madison, School of Medicine and Public Health, Madison, Wisconsin, United States; University of Wisconsin-Madison, School of Nursing, Madison, Wisconsin, United States; University of Wisconsin-Madison, School of Medicine and Public Health, Madison, Wisconsin, United States; University of Wisconsin-Madison, Madison, Wisconsin, United States

## Abstract

Nursing home (NH) regulations mandate resident and family engagement in care planning. Additionally, our previous research indicated residents/families want to be involved in care decisions. Care conferences are an important opportunity for residents/families to engage in decision-making. However, there is a lack of guidance or tools to support their engagement and a dearth of research on the most effective approaches to doing so. This study aimed to pilot test a family engagement intervention using resident, family, and staff communication tools that refocus the agenda of care conferences to topics identified as important by residents/families. The feasibility of the intervention was evaluated using a pre/post-implementation design and a preliminary analysis of effects was performed. A total of 16 (n=8 pre; n=8 post-implementation) care conferences (average length ~22min) were observed, audio-recorded, and coded using the Roter Interaction Analysis System and our prior engagement coding schemas. Frequencies of engagement outcomes were compared pre- and post-implementation using Mann-Whitney U and Fisher’s exact tests. Results suggest participants found the intervention valuable for care planning. The agenda and focus of care conferences also shifted to topics residents/families deemed important. Levels of engagement in relationship building and information exchange generally trended in a positive direction. Resident/family agenda setting was significantly higher after implementation (p=0.001). This study provides important insights into enhancing resident/family engagement in decision-making and suggests incorporating communication tools into care conferences may promote resident/family engagement. Future studies using randomized controlled trial designs and larger samples should be conducted to further evaluate effects.

